# Should helical tomotherapy replace brachytherapy for cervical cancer? Case report

**DOI:** 10.1186/1471-2407-10-637

**Published:** 2010-11-23

**Authors:** Chen-Hsi Hsieh, Ming-Chow Wei, Yao-Peng Hsu, Ngot-Swan Chong, Yu-Jen Chen, Sheng-Mou Hsiao, Yen-Ping Hsieh, Li-Ying Wang, Pei-Wei Shueng

**Affiliations:** 1 Department of Radiation Oncology, Far Eastern Memorial Hospital, Taipei, Taiwan; 2 Departments of Obstetrics and Gynecology, Far Eastern Memorial Hospital, Taipei, Taiwan; 3 Departments of Anatomic Pathology, Far Eastern Memorial Hospital, Taipei, Taiwan; 4 Institute of Traditional Medicine, School of Medicine, National Yang-Ming University, Taipei, Taiwan; 5 Department of Radiation Oncology, Mackay Memorial Hospital, Taipei, Taiwan; 6 Department of Medical Research, Mackay Memorial Hospital, Taipei, Taiwan; 7 Graduate Institute of Sport Coaching Science, Chinese Culture University, Taipei, Taiwan; 8 Department of Healthcare Administration, Asia University, Taichung, Taiwan; 9 Department of Radiation Oncology, National Defense Medical Center, Taipei, Taiwan; 10 School and Graduate Institute of Physical Therapy, College of Medicine, National Taiwan University, Taipei, Taiwan

## Abstract

**Background:**

Stereotactic body radiation therapy (SBRT) administered via a helical tomotherapy (HT) system is an effective modality for treating lung cancer and metastatic liver tumors. Whether SBRT delivered via HT is a feasible alternative to brachytherapy in treatment of locally advanced cervical cancer in patients with unusual anatomic configurations of the uterus has never been studied.

**Case Presentation:**

A 46-year-old woman presented with an 8-month history of abnormal vaginal bleeding. Biopsy revealed squamous cell carcinoma of the cervix. Magnetic resonance imaging (MRI) showed a cervical tumor with direct invasion of the right parametrium, bilateral hydronephrosis, and multiple uterine myomas. International Federation of Gynecology and Obstetrics (FIGO) stage IIIB cervical cancer was diagnosed. Concurrent chemoradiation therapy (CCRT) followed by SBRT delivered via HT was administered instead of brachytherapy because of the presence of multiple uterine myomas with bleeding tendency. Total abdominal hysterectomy was performed after 6 weeks of treatment because of the presence of multiple uterine myomas. Neither pelvic MRI nor results of histopathologic examination at X-month follow-up showed evidence of tumor recurrence. Only grade 1 nausea and vomiting during treatment were noted. Lower gastrointestinal bleeding was noted at 14-month follow-up. No fistula formation and no evidence of haematological, gastrointestinal or genitourinary toxicities were noted on the most recent follow-up.

**Conclusions:**

CCRT followed by SBRT appears to be an effective and safe modality for treatment of cervical cancer. Larger-scale studies are warranted.

## Background

It has been demonstrated that concurrent chemoradiation therapy (CCRT) followed by intracavity radiation is effective in the treatment of advanced cervical cancer [1]. Although external beam radiotherapy combined with brachytherapy is associated with high survival rates and low complication rates [2,3], patients with contraindications to brachytherapy, namely patients with unusual anatomic configurations of the pelvis or tumors, may benefit from higher doses of external beam irradiation [2]. However, studies have shown that external beam irradiation used throughout the treatment course for cervical cancer is associated with poor survival, poor local control, and a high incidence of side effects [3,4]. Molla et al. found that the use of intensity-modulated radiation therapy (IMRT) to deliver a final boost to areas at high risk for relapse in patients with endometrial or cervical cancer was feasible, well tolerated, and may be considered an acceptable alternative to brachytherapy [5]. Helical tomotherapy (HT), an image-guided IMRT, can deliver highly conformal dose distributions and provides an impressive critical organ sparing ability for cervical cancer [6]. Studies have shown that stereotactic body radiation therapy (SBRT), when using image-guided IMRT capable of delivering high doses of radiation in hypo-fractions, such as the HT system, is an effective and well-tolerated treatment for local control of tumors metastatic to the liver and lung [7,8]. Herein, we report on a patient with locally advanced cervical cancer that was treated with HT-guided SBRT rather than brachytherapy because the presence of an unusual anatomic configuration of the uterus was a contraindication to the use of brachytherapy.

## Case presentation

**Figure 1:**
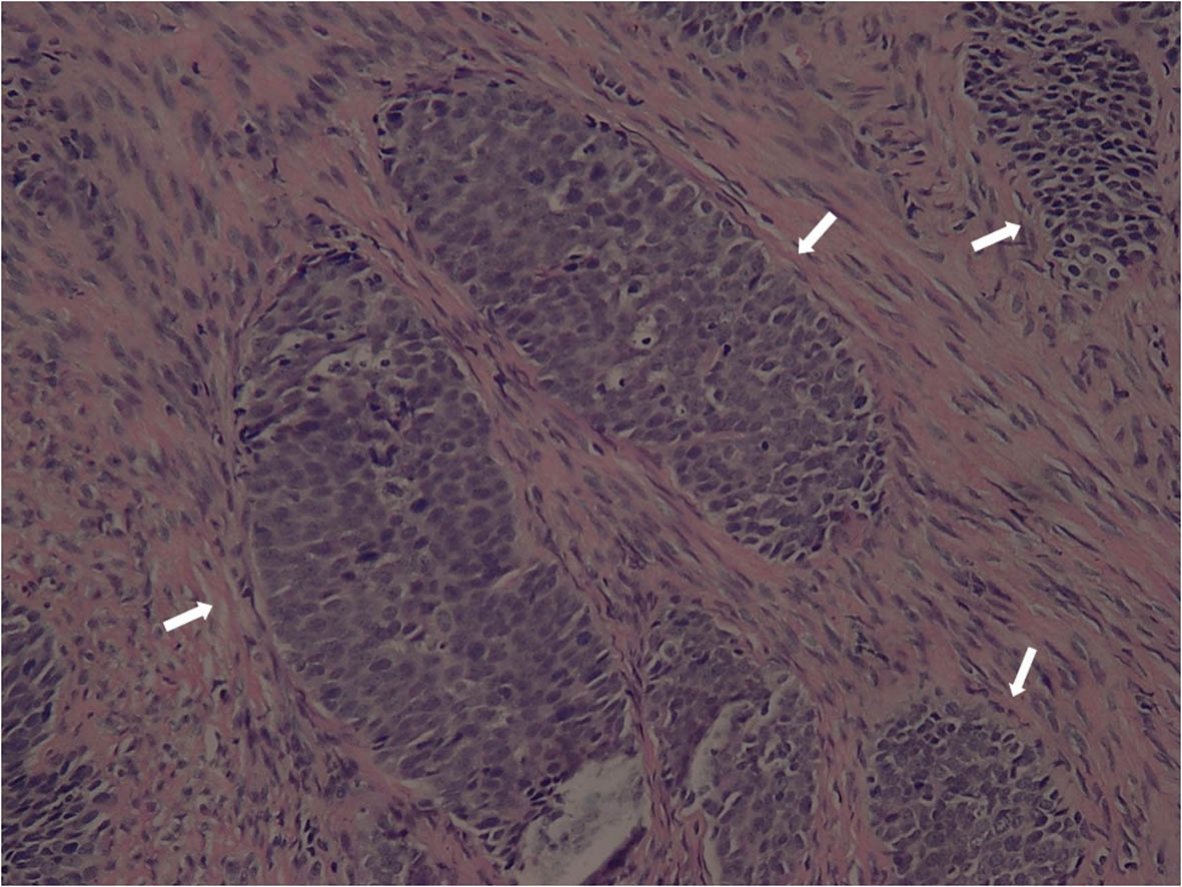
**Photomicrograph of cervical biopsy specimen before treatment shows nests of moderately differentiated squamous carcinoma cells invading deeply into the fibrous stroma (H-E 200X)**. The arrows indicate the tumor nests.

**Figure 2:**
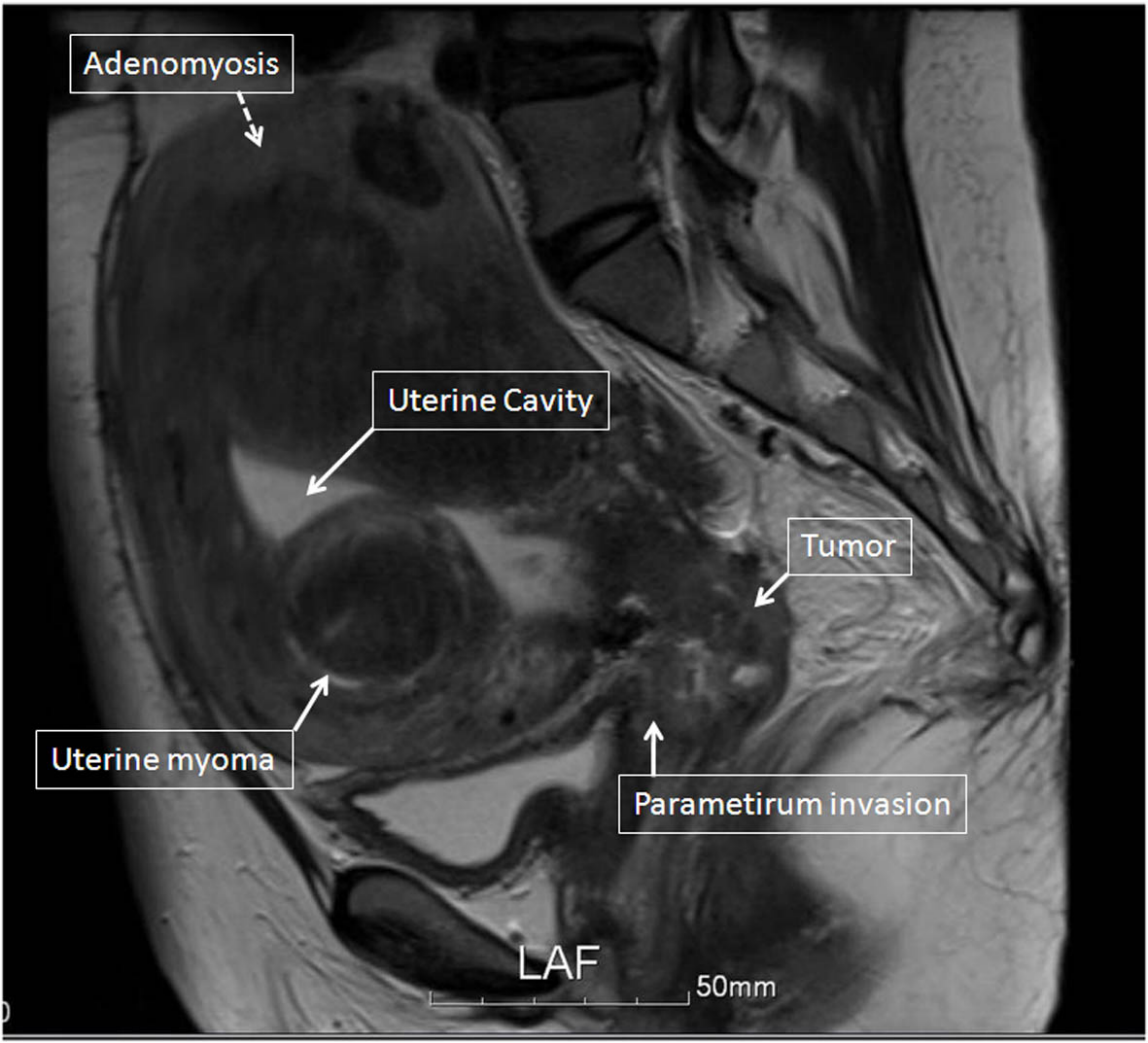
**MR image of the pelvis before treatment shows direct invasion of the cervical tumor into the right parametrium, bilateral hydronephrosis, and multiple uterine myomas**. The solid arrows indicate the parametrium invasion, the uterine myomas, the tumor, and the uterine cavity. The dotted arrow indicates adenomyosis.

**Figure 3:**
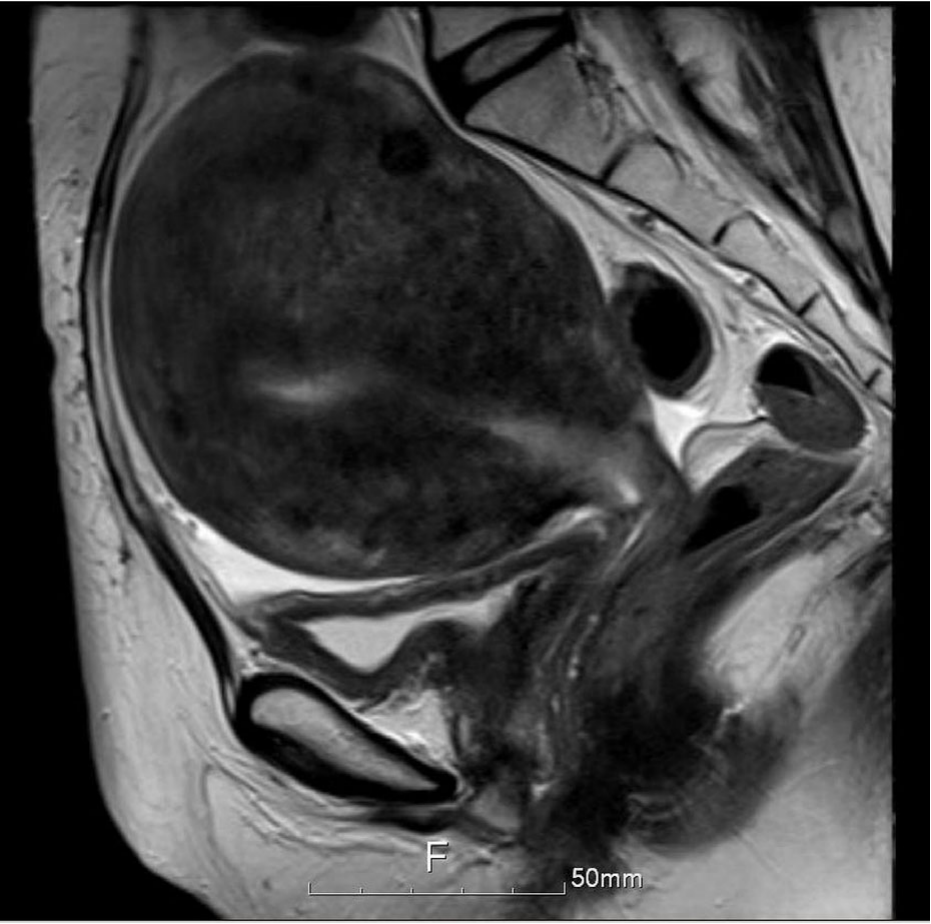
**Pelvic MRI after concurrent chemoradiation therapy and HT-guided SBRT shows multiple uterine myomas and adenomyosis without local recurrence or pelvic lymphadenopathy**.

**Figure 4:**
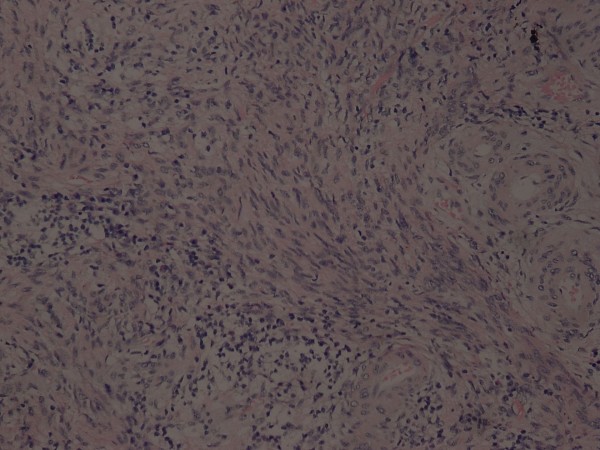
**Photomicrograph of cervical biopsy specimen taken after concurrent chemoradiation therapy followed by HT-guided SBRT shows only scattered nests of mononuclear inflammatory cells (H-E 200X)**.

**Figure 5:**
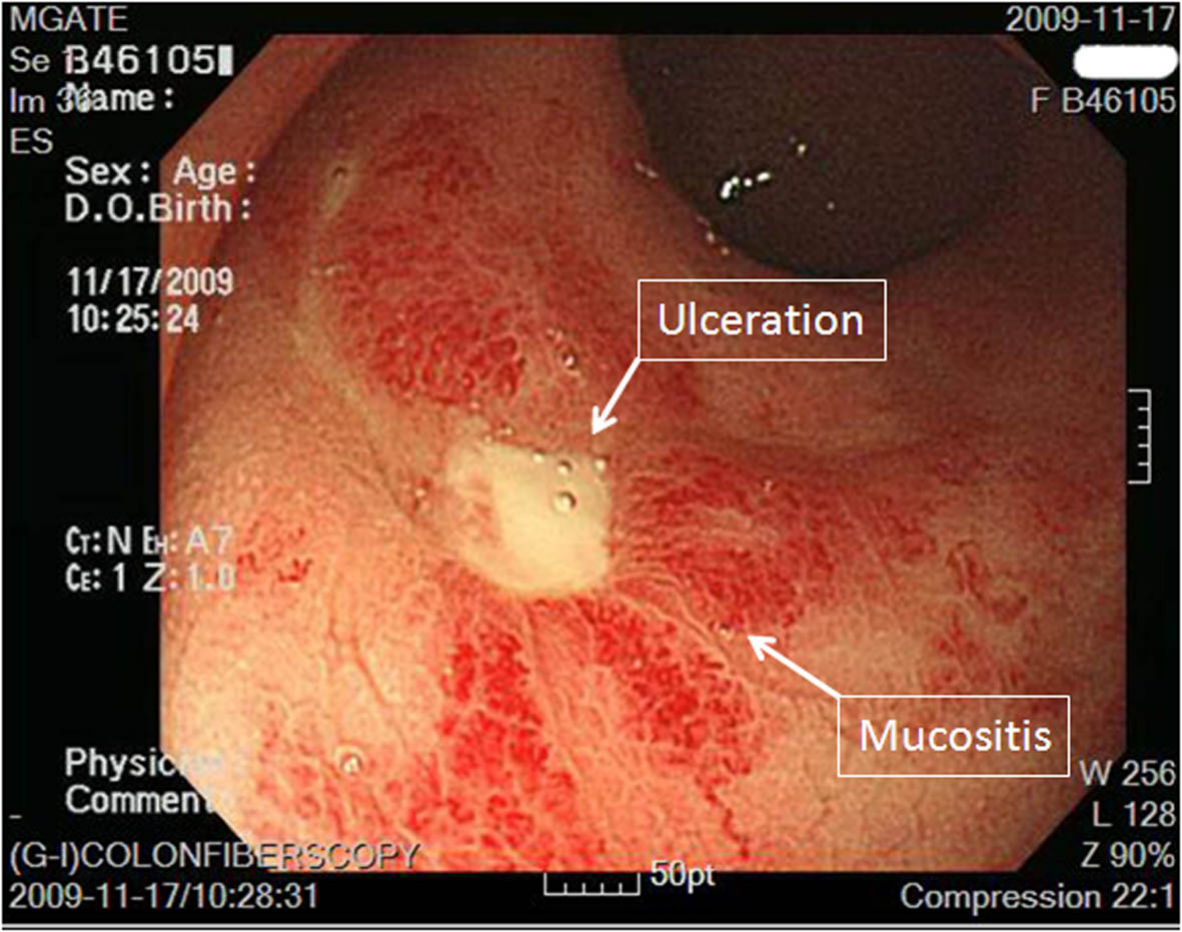
**At 14-month follow-up, ulceration and mucositis in the rectal area were noted**. The arrows indicate the ulceration and mucositis in the rectal area.

A 46-year-old woman presented with an 8-month history of abnormal vaginal bleeding. Cervical biopsy and Papanicolaou test results showed squamous cell carcinoma of the cervix (Figure 1). Magnetic resonance imaging (MRI) showed a cervical tumor with direct invasion of the right parametrium, multiple uterine myomas, and bilateral hydronephrosis (Figure 2). Laboratory test results revealed a BUN level of 21 mg/dL and a Creatinine level of 0.7 mg/dL. International Federation of Gynecology and Obstetrics (FIGO) stage IIIB cervical cancer was diagnosed. There was no evidence of distant metastasis at that time. The patient underwent CCRT. The major tumor and whole pelvis was treated with 54 and 48.5 Gy in 27 fractions over 6 weeks with simultaneous integrated boost techniques. The Pinnacle3 treatment planning system with 6- MV linear accelerator (Philips Healthcare, Madison, Wisconsin, USA) was used for treatment. Weekly cisplatin at a dose of 40 mg/m^2 ^was administered during external radiation for 5 weeks concurrent with radiotherapy. Because of the presence of multiple uterine myomas with bleeding tendency, the SBRT technique with 24 Gy delivered to primary tumor part with 0.7 cm margin as PTV in 6 fractions over one week was used in place of brachytherapy after receiving approval from the institutional ethics committee of the Far Eastern Memorial Hospital. The field width, pitch, and modulation factor (MF) used to optimize SBRT treatment were 2.5 cm, 0.32, and 3.0, respectively. Moreover, the 90% isodose surface covered between 95% and 98% of the planning target volume (PTV). Volumes of overdose exceeding 115% < 5% of the PTV were considered acceptable. Follow-up MRI taken one month after completion of treatment showed no evidence of tumor recurrence (Figure 3). However, at 6-week follow-up, the patient presented with several days of continuous vaginal bleeding accompanied by abdominal pain. The etiology of the bleeding and abdominal discomfort was believed to be due to the multiple uterine myomas. Therefore, a total abdominal hysterectomy was performed. Histopathologic examination revealed chronic inflammation of the cervix with no residual tumor (Figure 4). Toxicity of treatment was scored according to the Common Terminology Criteria for Adverse Events v3.0 (CTCAE v3.0). Only grade 1 nausea and vomiting during treatment were noted. At 14-month follow-up, controllable ulceration and mucositis were noted in the rectal area. (Figure 5) At the 22-month follow-up, no haematological, gastrointestinal, or genitourinary toxicities were noted. In addition, there was no evidence of fistula formation, local recurrence, or distant metastasis.

## Conclusions

External-beam irradiation could be an alternative to brachytherapy in cervical cancer patients with contraindications to external irradiation and brachytherapy [2]. IMRT and 3-dimensional conformal radiotherapy (3DCRT) are now widely used radiotherapy techniques for various cancers and have been shown to be acceptable alternatives to brachytherapy for the treatment of gynecologic malignancies [5,9].

**Figure 6:**
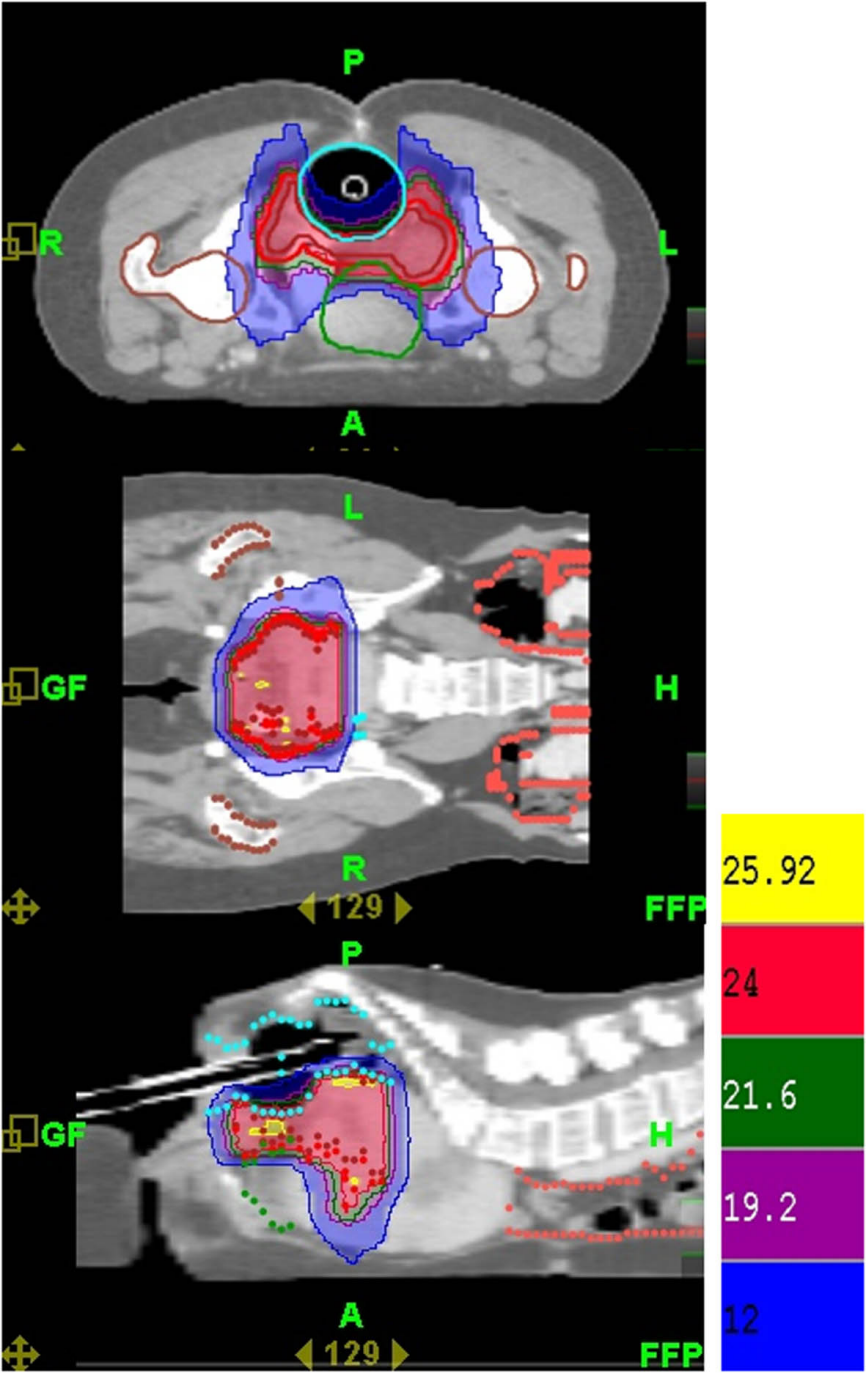
**The dose distribution of stereotactic body radiation therapy (SBRT) for the final boost for cervical cancer**.

HT-guided SBRT has been shown to be effective and well tolerated in patients with metastatic liver tumors [7], and in patients with small lung tumors [8]. In our patient, image-guided SBRT administered as a boost following CCRT resulted in a recurrence-free outcome without fistula formation at 22-month follow-up (Figure 3 and 4). However, at 14-month follow-up, the patient presented with lower GI bleeding (Figure 5). Retrospecting the planning, the conformal index [10] is 1.24. Additionally, the dose distribution has described in Figure 6. The mean dose of rectum is 45.5 Gy and the maximum dose of the rectum is 81 Gy where is close to the tumor and compatible to the bleeding area. The incidence of major late sequalae of RT for stages IIB and III of the cervix ranges from 10% to 15% [11]. Perez et al. [11] and Pourquier et al. [12] reported that with doses below 75 to 80 Gy delivered in limited volumes by a combination of external beam and intracavitary insertions with low dose rate (60 to 80 rad/hr), the incidence of grade 2 and 3 complications was less than 5%. However, with higher doses, the incidence of complications increased to 10% to 15%. In patients receiving total doses of 60 Gy to the rectum, more complications were noted [11]. Lower GI bleeding as a late complication of an external beam boost has been reported [2,5]. Although HT has the ability to accurately identify both the exact shape and location of the tumor so as to distribute the dose as close as possible to the margin around the target, the radiation oncologist needs to monitor the maximum doses to organs at risk (OARs) around the tumor in order to minimize, if not avoid, complications.

Uterine myomas are the most common neoplasms of the female pelvis, occurring in 20 - 25% of women of reproductive age [13] and the common symptoms were menorrhagia or metrorrhagia, or both [14-16]. It is the existence and level of bother of uterine fibroid symptoms that lead women to seek treatment, with the current standard of care being abdominal hysterectomy [17,18].

Symptoms of chronic radiation proctitis can manifest as mucous rectal discharge, diarrhea, urgency, pain, bleeding, and anemia. Radiation proctitis can be treated using steroid therapy [19,20], aminosalicylates [20], sucralfate enemas in combination with [21] or without [22] sulfasalazine, formalin [23,24], endoscopic Nd:YAG laser treatment [25,26], electrocoagulation, argon plasma coagulation [27,28], or hyperbaric oxygen [29,30]. However, the effectiveness of many of those therapeutic modalities has not been proven in controlled trials.

HT-guided SBRT appears to be an effective and safe alternative to brachytherapy for treatment of cervical cancer in patients with contraindications to that conventional treatment modality. Long-term follow-up is needed to confirm these preliminary findings. Radiation oncologists need to monitor the maximum doses to organs at risk around the tumor in order to avoid SBRT-induced complications.

## Consent

Written informed consent was obtained from the patient for publication of this case report and all accompanying images. A copy of the written consent is available for review.

## Competing interests

The authors declare that they have no competing interests.

## Authors' contributions

CHH and PWS carried out all CT evaluations, study design, target delineations and interpretation of the study. YPH carried out pathology study. CHH drafted the manuscript. MCW and SMH took care of cervical cancer patient. NSC participated in radiotherapy plan design. YJC, LYW and YPH gave advice on the work. All authors read and approved the final manuscript.

## Pre-publication history

The pre-publication history for this paper can be accessed here:

http://www.biomedcentral.com/1471-2407/10/637/prepub
